# Molecular Testing for Intrahepatic Cholangiocarcinoma: What, When, How?

**DOI:** 10.1007/s12029-026-01397-y

**Published:** 2026-01-15

**Authors:** Maryam Barsch, Elaine-Pashupati Dopfer, Anne Maria Schultheis, Michael Quante

**Affiliations:** 1 Clinic for Internal Medicine II, Gastroenterology, Hepatology, Endocrinology and Infectious Disease, University Hospital Freiburg, Freiburg, Germany; 2 Comprehensive Cancer Center Freiburg, Medical Center—University of Freiburg, Faculty of Medicine, University of Freiburg, Freiburg, Germany; 3 Institute for Surgical Pathology, Medical Center—University of Freiburg, Faculty of Medicine, University of Freiburg, Freiburg, Germany

**Keywords:** Intrahepatic cholangiocarcinoma, Biliary tract cancer, Molecular profiling, Targeted therapy, Next-generation sequencing, Precision oncology

## Abstract

**Purpose:**

Intrahepatic cholangiocarcinoma (iCCA) represents a biologically heterogeneous subgroup of biliary tract cancer (BTC) with a 5-year survival below 20%. Delayed diagnosis, intrinsic aggressiveness, and extensive intertumoral and intratumoral heterogeneity at the clinical, histopathological, and genomic level all contribute to this dismal outcome. Increasingly, treatment decisions in advanced iCCA depend on the identification of actionable molecular alterations, including FGFR2 fusions or rearrangements, IDH1 mutations, HER2 amplifications, NTRK fusions, and microsatellite instability (MSI-high) and/or mismatch repair deficiency (dMMR).

**Methods:**

A narrative review of the current literature was conducted, focusing on (i) what molecular alterations are clinically relevant today, (ii) when molecular profiling should be performed, and (iii) how testing should be technically implemented in routine clinical practice. Results iCCA, particularly the small-duct subtype, harbors a high prevalence of therapeutically actionable molecular alterations, in contrast to extrahepatic cholangiocarcinoma and gallbladder carcinoma. Early and comprehensive molecular profiling enables access to approved targeted therapies, molecularly stratified second-line treatments, and clinical trials. Combined DNA- and RNA-based next-generation sequencing, complemented by immunohistochemistry and in situ hybridization, provides the most reliable diagnostic framework.

**Conclusion:**

Molecular testing has become an essential component of modern iCCA management. Broad, early, and technically integrated molecular profiling—ideally performed at initial diagnosis and interpreted in an interdisciplinary (molecular) tumor board—is critical to fully realize the potential of precision oncology in BTC.

## Introduction

**Fig. 1:**
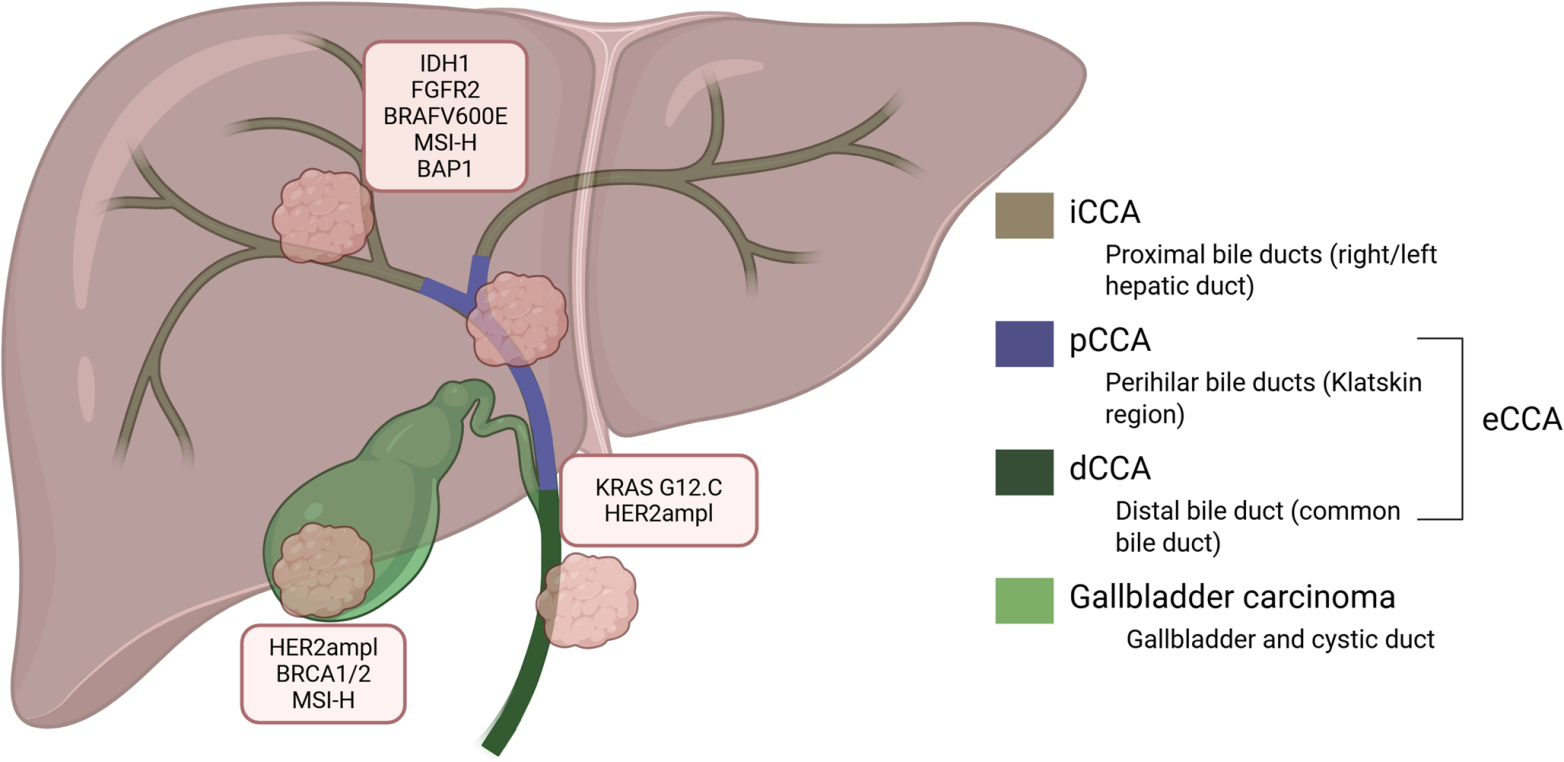
Anatomical and molecular heterogeneity of biliary tract cancers

Cholangiocarcinoma (CCA) refers to a heterogeneous group of epithelial tumors that arise from the biliary tract with cholangiocytic differentiation. Four major anatomical subtypes are recognized: intrahepatic cholangiocarcinoma (iCCA), perihilar cholangiocarcinoma (pCCA, “Klatskin tumors”), distal cholangiocarcinoma (dCCA), and gallbladder cancer. pCCA and dCCA can collectively be classified as extrahepatic CCA (eCCA) (Fig. 1). These subtypes differ not only in location and clinical characteristics, but also exhibit substantial molecular differences [1].

Schematic overview of the biliary tract illustrating the major anatomical subtypes of biliary tract cancer and their characteristic molecular alteration profiles. Intrahepatic cholangiocarcinoma (iCCA), arising from the intrahepatic bile ducts, is enriched for alterations such as IDH1 mutations, FGFR2 fusions/rearrangements, BRAF V600E mutations, BAP1 loss, and microsatellite instability–high (MSI-H). Perihilar cholangiocarcinoma (pCCA; Klatskin tumors), originating at the confluence of the right and left hepatic ducts, more frequently harbors KRAS alterations and ERBB2 (HER2) amplification. Distal cholangiocarcinoma (dCCA), arising from the extrahepatic bile duct (ductus choledochus), and gallbladder carcinoma show overlapping molecular features, including KRAS mutations, ERBB2 amplification, BRCA1/2 alterations, and MSI-H in a minority of cases. The figure highlights that biliary tract cancers represent biologically distinct entities rather than a single disease, with implications for molecular testing strategies and targeted therapy selection. This figure was created with Biorender.com, adapted from Barsch et al. [2]

Particularly iCCA, which is frequently of the histological small-duct type, harbors a notably high prevalence of therapeutically actionable genomic alterations such as *FGFR2*-fusions and *IDH1-*mutations (Fig. 1). In contrast, pCCA and dCCA, as well as gallbladder carcinoma, more commonly exhibit *KRAS*-alterations and *ERBB2-*/HER2 amplification/overexpression (Fig. 1) [3]. This heterogeneity is clinically relevant, as it determines which molecular alterations must be assessed, which testing modalities are most appropriate and whether patients are eligible for approved targeted treatments, off-label approaches, or clinical trials. The availability of multiple approved second-line targeted therapies, including agents directed against FGFR2 [4, 5], *IDH1-*mutations [6], *ERBB2*-/HER2 amplification/overexpression [7], MSI-high/dMMR [8], and tumor-agnostic therapies for *NTRK*-fusions [9], further underscores that CCA is not a single disease entity but a collection of diseases with distinct molecular subtypes (Fig. 1).

Accordingly, the central question that should guide current biliary oncology is “has comprehensive molecular profiling been performed, and was it done with the right method on the right specimen at the right time?” This review outlines a distinct molecular diagnostic algorithm.

## What to Test: Current Clinically Actionable Alterations in Cholangiocarcinoma

The molecular alterations outlined below represent the key actionable drivers in CCA, informing approved therapies, guideline-endorsed off-label use, or prioritization for clinical trial enrolment.

### *FGFR2*-Fusions/Rearrangements

**Fig. 2:**
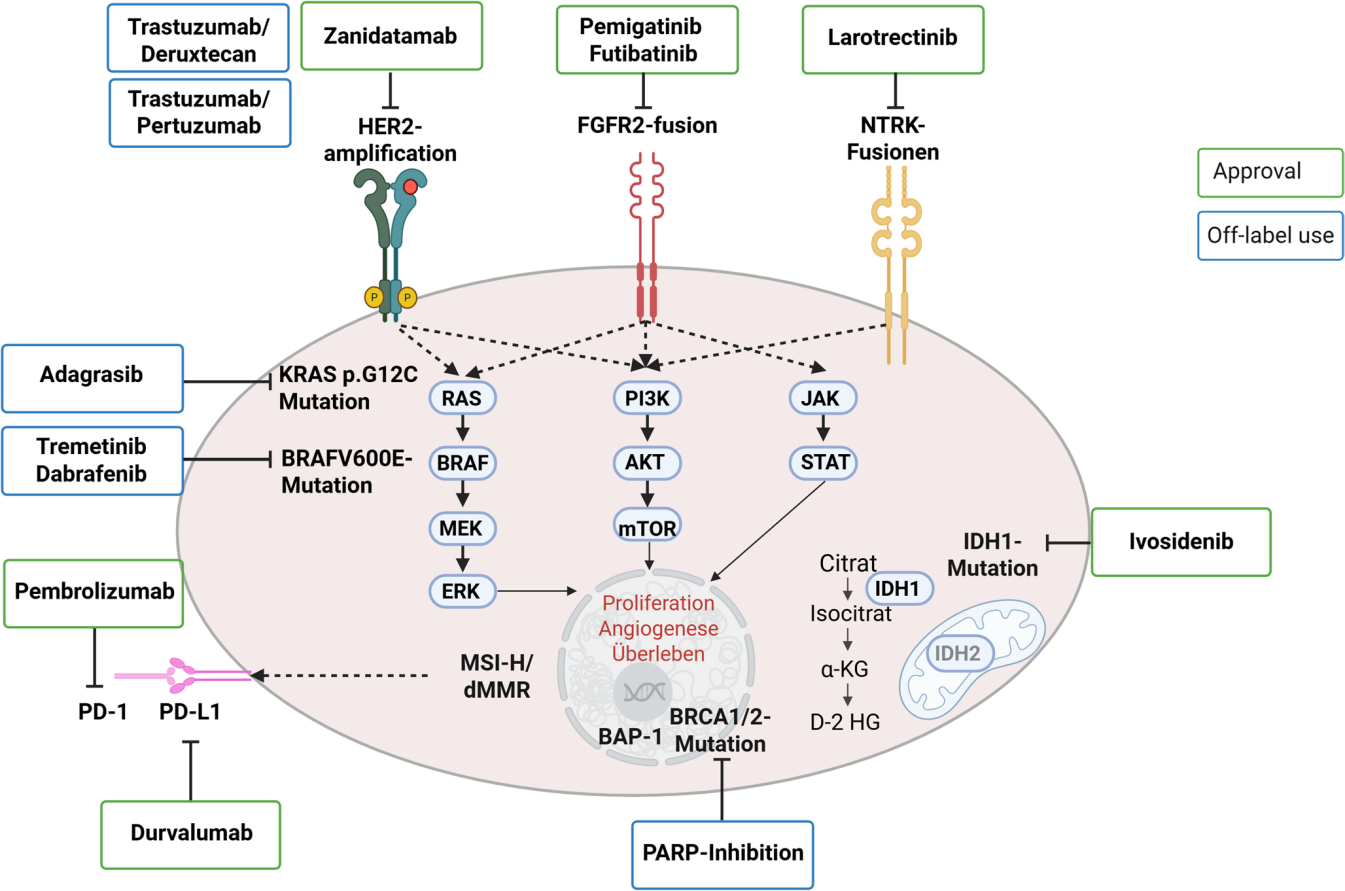
Key oncogenic signaling pathways and actionable molecular alterations in iCCA

FGFR2 is a receptor tyrosine kinase whose oncogenic activation typically arises through in-frame fusions resulting from chromosomal rearrangements that enhance dimerization or create ligand-independent signaling, thereby activating MAPK, PI3K–AKT, and JAK–STAT cascades (Fig. 2) [10].

Overview of major oncogenic signaling pathways and representative actionable molecular alterations in intrahepatic cholangiocarcinoma. Activating alterations in receptor tyrosine kinases and downstream effectors converge on core signaling cascades, including the RAS–RAF–MEK–ERK, PI3K–AKT–mTOR, and JAK–STAT pathways, promoting tumor cell proliferation, angiogenesis, and survival. Key alterations include FGFR2 fusions/rearrangements, IDH1 mutations leading to accumulation of the oncometabolite D-2-hydroxyglutarate, BRAF V600E and KRAS p.G12C mutations, mismatch repair deficiency/microsatellite instability–high (MMRd/MSI-H), and alterations in DNA damage repair genes such as BRCA1/2 and BAP1. Green boxes indicate molecular targets with approved therapies in biliary tract cancer or tumor-agnostic indications, while blue boxes indicate alterations currently addressed primarily through off-label use or clinical trials. The figure emphasizes the molecular diversity of iCCA and the rationale for comprehensive, pathway-oriented molecular profiling. This figure was created with Biorender.com, adapted from Barsch et al. [2]

These alterations occur in approximately 10–20% of small-duct iCCA and are far less common in eCCA or gallbladder carcinoma [11–14]. Clinically, *FGFR2-*fusions represent one of the most maturely validated targets in iCCA: In patients with *FGFR2*-fusion positive disease, the selective FGFR inhibitor Pemigatinib induced an objective response rate (ORR) of 37%, a median progression-free survival (PFS) of 6.9 months and a median overall survival (OS) of 21.1 months in the phase II FIGHT-202 trial [4]. In a similar population, the irreversible FGFR inhibitor Futibatinib achieved an ORR of 42%, a median PFS of 9.0 months and a median OS of 21.7 months in the phase II FOENIX-CCA2 study, with a median DOR of 9.7 months [5]. Hyperphosphataemia, driven by on-target interference with renal FGF2/3-FGFR1 signalling, is the most characteristic class toxicity of FGFR inhibitors, occurs in the majority of treated patients across agents, and requires systematic monitoring and management in routine practice [4, 5].

Given that more than 150 different *FGFR2-*fusion partners have been described and due to the marked heterogeneity of the genomic architecture of these fusions, fusion partner agnostic sequencing-based approaches capable of identifying yet unknown fusion partners are superior to FISH-based tests [10].

A major challenge with FGFR inhibition is the frequent development of acquired resistance, typically through polyclonal secondary kinase-domain mutations in *FGFR2*. At radiologic progression, re-biopsy or high-quality ctDNA analysis can help identify such resistance mutations and thereby inform subsequent therapeutic choices, including the rational use of next-generation inhibitors, such as irreversible FGFR inhibitors, within clinical trials or selected off-label settings.

Importantly, current approvals are restricted to tumors harboring *FGFR2*-fusions or rearrangements. For isolated *FGFR2* point mutations or amplifications, clinical efficacy data are limited, and their responsiveness to FGFR inhibition remains far less established than that of canonical *FGFR2*-fusions, highlighting the need for further research.

### *IDH1-M*utations

Pathogenic *IDH1-*mutations result in the accumulation of the oncometabolite 2-hydroxyglutarate, which disrupts normal epigenetic regulation and cellular differentiation. These alterations occur in approximately 10–20% of iCCAs and are particularly enriched in the histological small-duct subtype (Fig. 2). Their therapeutic relevance was demonstrated in the randomized phase III ClarIDHy trial, in which the IDH1 inhibitor ivosidenib significantly improved progression-free survival compared with placebo (median PFS 2.7 vs. 1.4 months; hazard ratio 0.37) [6]. Median OS numerically favored ivosidenib in the intent-to-treat population (10.3 vs. 7.5 months), and after statistical adjustment for the high rate of crossover from placebo to ivosidenib, the estimated median OS for the placebo group decreased to 5.1 months, supporting a clinically meaningful survival benefit [6]. However, radiographic tumor shrinkage was rare, with an ORR of about 2%; the disease control rate was driven predominantly by stable disease, indicating that IDH1 inhibition in this setting mainly achieves disease stabilization rather than radiological remission. From a diagnostic perspective, detection of *IDH1-*mutations is straightforward, as standard DNA-based targeted NGS panels reliably identify the canonical hotspot variants, and RNA-level analysis is not required.

### HER2 (ERBB2) Amplification and Overexpression

HER2 (ERBB2) is an established oncogenic driver in various tumor entities. Aberrant HER2 signaling activates canonical RAS–RAF–MEK–ERK and PI3K–AKT–mTOR pathways and thereby promotes tumor cell proliferation and survival (Fig. 2). *ERBB2*/HER2 amplification and/or overexpression are particularly enriched in extrahepatic CCA (eCCA) and gallbladder carcinoma, where reported rates range roughly between 10% and 27%. While around 5% of large duct iCCA show *ERBB2* amplification, classic small-duct iCCA is even less frequently HER2 driven. From a diagnostic perspective, *ERBB2*/HER2 assessment must encompass both protein and gene level: IHC is used to semi-quantitatively score membranous overexpression (0/1+/2+/3+) as initially established for gastric cancer [15], IHC 2 + cases require confirmatory in-situ-hybridization, typically fluorescence in situ hybridization (FISH) for *ERBB2* amplification. DNA-based NGS panels can in parallel identify *ERBB2* point mutations and copy-number variants. In contrast, HER2-low status (IHC 1 + or 2 + without amplification) has not translated into a robust therapeutic signal in BTC; high-level overexpression and/or gene amplification remain the clinically relevant actionable target, which is supported by several lines of evidence. The phase IIa MyPathway basket study showed that dual blockade with trastuzumab plus pertuzumab in previously treated HER2-overexpressed or amplified metastatic BTC achieved a confirmed ORR of 23% (9/39 patients) with an acceptable toxicity profile, providing an early proof of concept for HER2 targeting in this setting [16]. HER2-directed antibody–drug conjugates (ADCs) have further diversified the therapeutic options: in the single-arm phase II HERB trial, trastuzumab deruxtecan (T-DXd) produced a confirmed ORR of 36.4% in HER2-positive BTC, with a median progression-free survival of approximately 4.4 months and OS of around 7 months, whereas the exploratory HER2-low cohort showed a much more modest response rate of 12.5% [17]. These findings underscore that high-level HER2 expression is the clinically relevant therapeutic threshold in BTC. Complementary activity has also been demonstrated with small-molecule HER2 inhibition: in the SGNTUC-019 basket trial, tucatinib plus trastuzumab achieved an ORR of about 47% and a median progression-free survival of 5.5 months in pretreated HER2-positive BTC [18].

The most practice-changing data, however, come from zanidatamab, a bispecific antibody targeting two distinct HER2 epitopes. In the global, multicentre, single-arm phase IIb HERIZON-BTC-01 study, patients with *ERBB2*-amplified, unresectable or metastatic BTC who had progressed after gemcitabine-based therapy and whose tumors overexpressed HER2 (IHC 3 + or 2 + with amplification) received zanidatamab monotherapy [7]. The trial reported a confirmed ORR of 41% in the overall population, with additional exploratory data (from non–peer-reviewed sponsor and conference reports) indicating a ORR of about 52% among IHC 3 + tumors, a median duration of response of 14.9 months, a median PFS of 5.5 months (7.2 months in IHC 3 + disease) and a median OS of approximately 15.5 months with longer follow-up, alongside a manageable safety profile dominated by diarrhea and infusion-related reactions [7]. These results led to accelerated U.S. Food and Drug Administration (FDA) approval in November 2024 for previously treated unresectable or metastatic HER2-positive BTC defined as IHC 3+, and conditional authorization in the European Union in 2025, formally establishing zanidatamab as the first HER2-specific, disease-focused standard option in this setting and effectively elevating HER2 to a “must-test” biomarker in advanced BTC. A first-line phase II study (NCT03929666) combining zanidatamab with gemcitabine/cisplatin in HER2-positive BTC is ongoing, underlining the effort to move HER2-targeted therapy earlier in the treatment algorithm.

Taken together, current evidence and regulatory approvals converge on a consistent message: in BTC, HER2 is no longer an exploratory marker, but a clinically relevant therapeutic target that requires reliable testing with high-quality IHC, FISH in borderline (IHC 2+) cases, and supportive NGS. Treatment decisions should be based on unequivocal HER2 positivity (IHC 3 + and/or *ERBB2*-amplification), further repeated testing over disease progression could be considered.

### MSI-High/Mismatch Repair Deficiency (dMMR)

Defective mismatch repair (dMMR) leads to the accumulation of insertion–deletion mutations within microsatellite regions, generating a high neoantigen load and increased immunogenicity (Fig. 2). Although dMMR/microsatellite instability–high (MSI-high) status is rare in BTC, occurring in only about 1–2% of cases [11], it has major therapeutic implications, as MSI-high tumors show a high likelihood of deriving durable benefit from immune checkpoint inhibitior (ICI) as monotherapy (Fig. 2) [8, 19, 20]. Diagnosis can be established either by immunohistochemistry for the four MMR proteins (MLH1, MSH2, MSH6, PMS2) and by PCR- or NGS-based MSI assays [21]. Because MSI status may justify early integration of ICI, its identification can meaningfully influence both first- and second-line treatment planning; however, evidence for ICI after prior chemo-immunotherapy remains limited, and current guidelines do not offer a definitive recommendation for re-treatment in this setting [12, 22].

### *NTRK*-Fusions

Gene fusions involving *NTRK1*, *NTRK2* or *NTRK3* result in constitutively active TRK kinases that drive oncogenic signaling and are thus often mutually exclusive with other oncogenic diver mutations. Although such events are exceedingly rare in iCCA, with a prevalence well below 1% [12], these alterations are clinically meaningful because TRK inhibitors can induce high ORRs across a broad range of solid tumors, leading to tumor-agnostic approvals based on fusion status alone (Fig. 2) [9]. Given the extreme diversity of possible fusion partners, partner-agnostic hybrid-capture NGS with RNA-based fusion detection is considered the most reliable first-line method for identifying *NTRK*1–3-fusions in BTC. Pan-TRK IHC may serve as a preliminary screening approach, but its specificity is limited and it may both under-detect and over-call TRK expression in biliary epithelium; therefore, confirmatory NGS remains essential whenever an actionable fusion is suspected [23, 24].

### BRAF V600E

BRAF V600E leads to constitutive activation of MAPK signaling and occurs in approximately 3–6% of BTC (Fig. 2) [11]. Although uncommon, this alteration is clinically relevant because dual BRAF/MEK inhibition-most notably with dabrafenib plus trametinib-has produced meaningful ORR in BRAF V600E-mutant solid tumors, including iCCA, and is therefore frequently considered as an off-label treatment option in this setting (Fig. 2) [25]. Detection of this mutation is straightforward, as standard DNA-based hotspot NGS assays reliably identify BRAF V600E and do not require RNA-level analysis.

### KRAS p.G12C and Other *KRAS* Alterations

*KRAS* mutations drive MAPK pathway activation and are considerably more frequent in eCCA and gallbladder carcinoma than in iCCA, with KRAS p.G12C representing only a small fraction of these alterations (Fig. 2). Although single-agent KRAS G12C inhibitors have demonstrated clinical activity in other tumor types [26] and are increasingly considered off-label in selected patients with KRAS p.G12C-mutant BTC their efficacy as monotherapy remains limited. Early pooled phase I/II data with KRAS G12C inhibition, including small BTC cohorts, have reported encouraging response signals, but these findings remain exploratory and require confirmation in larger, disease-specific studies [27]. Standard DNA-based hotspot NGS reliably detects all clinically relevant *KRAS*-variants. Looking ahead, emerging pan-KRAS inhibitors under clinical investigation may broaden therapeutic opportunities by targeting a wider spectrum of *KRAS*-mutations beyond G12C. Rational combination strategies, which have extended PFS in other malignancies [28] are likewise being explored and may ultimately prove more effective than monotherapy in *KRAS*-mutant BTC.

### Other Emerging Targets

In addition to well-validated biomarkers, a number of less frequent molecular alterations have emerged as potentially actionable in BTC, though clinical data remain limited. These include *RET*-fusions [29], *NRG1*-fusions [30], *MDM2-*amplification [31] and germline *BRCA1/2*-variants. While each of these alterations offers a biologically plausible therapeutic target (e.g., RET inhibitors for *RET*-fusion tumors, pan-ERBB inhibition for *NRG1*-fusion cancers, MDM2 inhibitors for *MDM2*-amplifications and PARP inhibition for *BRCA1/2*-mutations), to date evidence in BTC is largely limited to preclinical data, case reports or very small patient series. These targets are still investigational and should be addressed through clinical trials or molecular tumor board–recommended off-label use.

## When to Test: the Clinical Imperative of Early Molecular Profiling

**Fig. 3:**
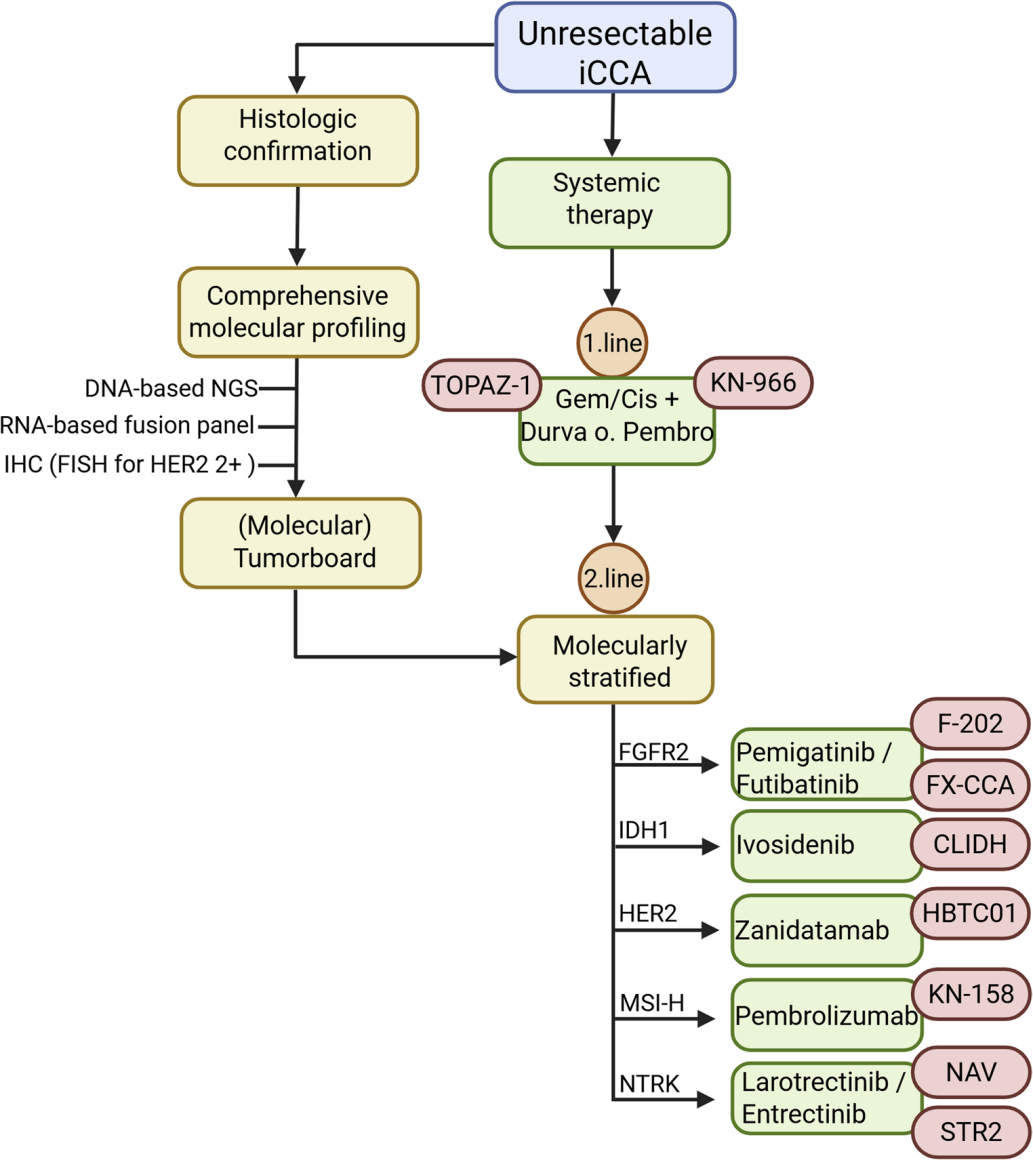
Pragmatic molecular diagnostic and treatment algorithm for unresectable iCCA

BTC is frequently diagnosed at an advanced stage. The disease is often clinically silent until biliary obstruction, cholestasis, jaundice, constitutional symptoms (fever, night sweats, weight loss), or metastatic spread occur. Especially iCCA may remain radiologically occult until large, fibrotic, mass-forming lesions are present in the liver, whereas pCCA and dCCA may first present through obstructive jaundice. Unlike hepatocellular carcinoma (HCC) (where imaging plus cirrhosis can establish the diagnosis), iCCA requires histological confirmation. Tissue can be obtained by image-guided core needle biopsy of a liver lesion, direct cholangioscopy-guided biopsies in selected intrahepatic lesions, or a surgical specimen (in potentially resectable disease) (Fig. 3) [12].

Proposed diagnostic and therapeutic workflow for patients with unresectable intrahepatic cholangiocarcinoma. Following histologic confirmation, comprehensive molecular profiling should be performed early, ideally at initial diagnosis or during first-line systemic therapy. First-line treatment consists of gemcitabine/cisplatin combined with immune checkpoint inhibition, based on the TOPAZ-1 and KEYNOTE-966 trials. Molecular results guide second-line, molecularly stratified treatment, including FGFR inhibition for FGFR2 fusions/rearrangements, IDH1 inhibition for IDH1-mutant tumors, HER2-directed therapy for ERBB2-amplified disease, immune checkpoint inhibition for MSI-H/MMRd tumors, and TRK inhibition for NTRK fusion–positive cancers. Molecular findings may be discussed in an interdisciplinary (molecular) tumor board, particularly for off-label treatment decisions or prioritization for clinical trial enrollment. The algorithm illustrates the integration of “what to test,” “when to test,” and “how to test” to enable timely access to precision oncology strategies in iCCA. This figure was created with Biorender.com.

Because many patients are unresectable at first presentation, systemic therapy is the foundation of current management (Fig. 3). First-line systemic treatment in fit patients is gemcitabine/cisplatin in combination with an ICI (durvalumab [32] or pembrolizumab [33]), and second-line options now include multiple targeted monotherapies for the above discussed defined genomic alterations (e.g. *FGFR2*-fusion → pemigatinib or futibatinib; *IDH1*-mutation → ivosidenib; MSI-high → pembrolizumab; *HER2*-amplification → zanidatamab; *NTRK*- fusion → entrectinib/larotrectinib) (Fig. 3).

This has two immediate diagnostic consequences: molecular profiling cannot be deferred ‘until progression,’ because many patients will never reach second line, and even for those who do, actionable results must already be available at the time second-line therapy is considered. Delaying testing until progression risks losing critical time, as tissue processing, molecular analysis, expert interpretation, and in some cases off-label authorization procedures can be time-consuming; therefore, profiling should be completed as early as possible in the disease course.

In other words: for iCCA, ‘test early’ is not an academic slogan but a survival-relevant imperative, as molecular profiling at initial diagnosis or at the latest during first-line therapy is essential to enable guideline-directed, molecularly stratified treatment in the second line and to identify additional off-label therapeutic opportunities.

## How To Test: Methodological Aspects and Practical Pitfalls

### DNA-based Targeted NGS Panels

DNA-based NGS testing performed on formalin-fixed paraffin-embedded (FFPE) tissue offers substantial advantages in the molecular diagnostic work-up of BTC. Its robustness on routinely processed clinical specimens enables reliable detection of single-nucleotide variants (SNVs), small insertions and deletions (indels), CNVs (e.g., *ERBB2*-amplifications), and a selection of structural variants. For canonical hotspot mutations, such as those in *IDH1*, *BRAF* V600E, *KRAS* (e.g., p.G12C), *TP53*, *ARID1A* or *BAP1*, DNA-based targeted panels are typically sufficient, reproducible and efficient, facilitating rapid turnaround in clinical practice [34–36].

However, this approach has known limitations that can compromise sensitivity and comprehensiveness. First, targeted DNA-only panels may fail to detect gene fusions [37, 38]. Consequently, *FGFR2*-fusions/rearrangements may be undercalled. Second, DNA-based NGS-testing does not directly assess protein overexpression (e.g., HER2 IHC) or functional consequences of mismatch repair deficiency (MMR loss), which require separate methodologies (IHC, FISH, MSI assays). Thus, reliance on DNA sequencing alone may miss clinically actionable alterations or lead to false-negative evaluations in cases where structural variation, gene amplification, or protein-level dysregulation drive oncogenesis [37].

### RNA-Based Fusion Profiling/Combined RNA–DNA Hybrid Capture

RNA-based fusion profiling-especially when combined with DNA sequencing in a hybrid-capture panel plays a pivotal role in the molecular work-up of BTC. The major strength of this approach is its ability to detect expressed in-frame fusions independently of known fusion partners, providing direct evidence that a chimeric oncogenic transcript is present and active [10]. This makes it the gold standard for identifying clinically relevant fusions such as *FGFR2*-rearrangements or fusions involving *NTRK*1–2 or rare partners such as *NRG1*, which often escape detection in DNA-only assays or targeted single-gene tests [37].

Indeed, for *FGFR2*-fusions combined RNA/DNA sequencing ensures reliable detection regardless of the fusion partner, an essential step to select patients for FGFR-directed therapy [10]. In the molecular biology of FGFR2, oncogenic activation is frequently driven by intrachromosomal rearrangements or loss of regulatory domains, resulting in ligand-independent dimerization and constitutive activation of downstream signaling pathways (e.g., MAPK, PI3K-AKT), which underlines the clinical relevance of identifying any *FGFR2-*fusion [39].

However, RNA-based fusion testing also has limitations. High-quality, sufficiently abundant RNA is required, a challenging demand in clinical routine, especially when using FFPE specimens. RNA is more labile than DNA, small, hypocellular biopsies, heavily cauterized tissue, or cytology specimens (e.g., brushings) may not yield enough intact RNA for reliable fusion detection. Consequently, negative RNA fusion results in such samples cannot definitively exclude a fusion-driven tumor [10, 38].

As a practical recommendation, when tissue is limited, clinicians and pathologists should prioritize sending the sample for a combined DNA + RNA NGS panel, instead of sequentially exhausting tissue on multiple individual single-gene assays. This maximizes the chance of detecting all relevant alterations, (SNVs, copy-number changes, and fusions) in a single test.

### Immunohistochemistry (IHC)

Immunohistochemistry (IHC) remains a key component of the diagnostic work-up for cholangiocarcinoma, particularly when evaluating small biopsies. A typical cholangiocellular immunophenotype includes CK7 positivity, frequent CA19-9 expression, and the absence of hepatocytic markers such as HepPar-1, which helps to distinguish iCCA from HCC [40, 41]. HER2 assessment relies on membranous IHC staining, interpreted using scoring systems analogous to those established in gastric cancer, and is essential for identifying tumors with HER2 overexpression or amplification [7, 42]. Furthermore, screening for mismatch repair deficiency requires IHC evaluation of the four MMR proteins (MLH1, MSH2, MSH6, PMS2), which is a sensitive and widely accessible approach for detecting dMMR in BTC [43].

Although IHC is rapid, cost-effective, and feasible even on minimal tissue samples, it has important limitations. In particular, IHC cannot reliably detect oncogenic gene fusions, such as *FGFR2*- or *NTRK*-rearrangements, nor can it differentiate between wild-type FGFR2 protein and fusion-derived chimeric proteins [10]. For these alterations, RNA-based fusion profiling or hybrid-capture NGS approaches are required to achieve accurate partner-agnostic detection.

### In Situ Hybridization (CISH/FISH)

In-situ hybridization techniques, such as CISH and FISH, remain valuable in specific diagnostic scenarios. For HER2, CISH/FISH is routinely used to confirm *ERBB2-*amplification in tumors with equivocal (IHC 2+) staining [42]. For *FGFR2*, break-apart CISH/FISH serves as a practical fallback when RNA- or DNA-based hybrid capture sequencing cannot be performed due to very limited tissue, as it can detect rearrangements in as few as 50–100 tumor cells [10]. However, given the known technical properties of FISH, this technique has notable limitations: it can confirm the presence of a rearrangement but does not identify the fusion partner, and thus cannot fully characterize complex structural variants. While this is generally acceptable for *FGFR2*, because available inhibitors are partner-agnostic, CISH/FISH is inherently not scalable to a large number of genomic targets. As such, it should be viewed as a targeted salvage tool, rather than a comprehensive molecular screening method.

## From What To when To How: Building a Practical Molecular Diagnostic Algorithm

Putting what, when, and how together, the following working algorithm for routine care can be proposed. First, the what: every patient with advanced CCA should undergo testing for the full set of clinically actionable alterations, most prominently *FGFR2* fusions/rearrangements, *IDH1*-mutations, *ERBB2*/HER2 amplification/overexpression, MSI-high/dMMR, *NTRK*-fusions, *BRAF* V600E, *KRAS* p.G12C, and selected emerging but still investigational targets (e.g., *RET*-fusions, *NRG1*-fusions, *MDM2*-amplification, *BRCA1/2*-alterations). This panel reflects the alterations that currently guide approved targeted therapies, strongly guideline-supported off-label options, or prioritization for clinical trial enrolment.

Second, the when: comprehensive molecular profiling must be performed as early as possible, ideally at initial diagnosis of unresectable or metastatic disease or no later than during first-line systemic therapy (Fig. 3). Delaying molecular testing until progression compromises clinical decision-making, many patients decline before second-line therapy becomes feasible, and because molecular results and, when applicable, off-label authorizations must already be available when second-line treatment is planned.

Third, the how: the diagnostic backbone should be a combined DNA–RNA NGS panel, which enables simultaneous detection of point mutations, indels, copy-number alterations, and critically partner-agnostic expressed gene fusions such as *FGFR2*, *NTRK*1–3, or *NRG1*. This should be complemented by IHC for HER2 (with FISH for IHC 2 + cases) and IHC for MMR proteins (MLH1, MSH2, MSH6, PMS2) to screen for MSI-high/dMMR.

Finally, all molecular findings should be reviewed in a (molecular) tumor board, which recommends established targeted therapy when available, determines eligibility for clinical trials, and where appropriate considers off-label targeted strategies (Fig. 3). At disease progression, repeated biopsy or high-quality ctDNA analysis may be used to identify resistance mutations, particularly in FGFR2-driven disease, and to re-enter the diagnostic–therapeutic algorithm.

## Challenges and Outlook

Despite substantial advances in molecular diagnostics, translating precision oncology into routine care for CCA remains challenging. Tissue scarcity persists as a major barrier, since small biopsies, brushings, or fine-needle aspirations frequently provide insufficient material for both, reliable histopathologic evaluation and comprehensive DNA/RNA-based molecular profiling. Turnaround time also remains critical, as molecular results must be available early enough to shape first- and second-line treatment decisions. Access and reimbursement constraints further complicate implementation, as hybrid-capture DNA/RNA sequencing is not universally supported, prompting reliance on sequential single-gene assays that risk missing partner-agnostic fusions.

Even with meaningful advances in molecular medicine, the overall clinical prognosis of iCCA remains very limited. Resistance to targeted therapy limits the durability of responses and highlights the need for longitudinal molecular monitoring, including ctDNA-based approaches, to anticipate resistance and inform therapeutic sequencing.

Moreover, recurrence rates of iCCA remain high even after resection with curative intent. Recent perioperative data illustrate that prognostic limitations may be modifiable. The GAIN study, presented at ASCO 2025, suggested that neoadjuvant gemcitabine/cisplatin can increase the likelihood of R0 resection and may improve survival in resectable iCCA, despite its early termination and small sample size [44]. These findings challenge the historic paradigm of immediate surgery and raise the possibility that neoadjuvant strategies might further improve outcomes. As iCCA is increasingly recognized as a group of molecularly defined subtypes rather than a single disease entity, a key future question is whether neoadjuvant treatment could also incorporate targeted or immunotherapeutic agents in biomarker-selected populations, such as FGFR2-directed therapy for *FGFR2*-fusion-positive tumors or HER2-directed treatment for *ERBB2*-amplified disease. These concepts remain investigational but represent a logical extension of precision oncology into the curative-intent setting.

Altogether, these limitations underscore the critical role of early, comprehensive molecular profiling at diagnosis as a foundation for developing future strategies to improve long-term outcomes in iCCA.

## Data Availability

No datasets were generated or analysed during the current study.
